# A Manual of Percussion and Auscultation

**Published:** 1876-10

**Authors:** 


### Editorial.

### BIBLIOGRAPHICAL.

A Manual of Percussion and Auscultation; of the Physical Diagnoses of the Lungs and Heart, and of Thoracic Anurism. By Austin Flint, M.D., Professor of the Principles and Practice of Medicine, and of Clinical Medicine in the Bellevue Hospital Medical College, etc., etc. Philadelphia: Henry C. Lea. 1876.

This hand-book of two hundred and fifty pages, by the highest modern authority on the physical signs of disease will be read with profit by student and practitioner of medicine. Dr. Flint's long course of study and teaching connected with the diagnosis of pectoral diseases, has carried him to unusual proficiency in this very useful and much neglected department of medical science. A sad want of information on this subject exists among medical men generally, and we hope the work will have a liberal run in the profession.

